# The Age of First Menstruation in the United States*Read before the American Gynecological Section, Chicago, May, 1901.

**Published:** 1901-09

**Authors:** G. J. Engelmann

**Affiliations:** Boston


For Texas Medical Journal.

### The Age of First Menstruation in the United States.*

BY G. J. ENGELMANN, M. D., BOSTON.

The period of pubértal development is an important epoch in girl life, as the crest of one of the three great waves of functional

*Read before the American Gynecological Section, Chicago, May, 1901.

activity, during which susceptibility to morbid influences is at its highest; much of woman's functional irregularity and suffering is due to neglect at this time, hence, the pubertal period claims the attention of the physician as a promising field for preventive gynecology.

To the physiologist and ethnologist the age of first menstruation in the United States, but little studied as yet, is of interest, as the extent of territory, with its variety of climate and the many races presented, offers exceptional advantages for the solution of still open problems as to the causes which influence pubertal development, which hasten or retard the appearance of the menstrual flow.

Over ten thousand observations as to the time of first menstruation of American-born women, many with reference to points never before investigated, here or elsewhere, give me ample material for an authoritative solution of the questions involved. These observations, from my own practice and that of helpful friends, are many, and the identity of results obtained in far distant points, Montreal and New Orleans, St. Louis, Cincinnati and Boston, vouches for their correctness; furthermore, they are corroborated by all previous records, a total of six thousand, in such points as these may cover.

The mean age of first menstruation in this country is 13.9 or 14 years, the same in the United States and Canada. Climatic differences in no wise influence pubertal development within the bounds of the North American continent; the American born, be they of American (14.1), German (14.5) or French (13.6) parentage of the same class, attain puberty at the same age in Montreal, St. Louis and Boston; the negro does not vary (14.05) whether in New Orleans or St. Louis. The greatest variation caused by the extremes of all influences is one year, from 13.5 in the girl of highest refinement and education to 14.5, which is the period for the American born of the laboring classes of German and Irish parentage; in other countries the difference between the extremes of social classes is from two to three years.

Refinement, education, city life, nerve stimulation, determine precocious puberty; ignorance, poverty and manual labor retard; social status in itself means very little; heredity and race have a slight determining influence.

The American born are more precocious than the women of other countries in the same zone; 14 is the age of puberty in the United States and Canada, 15.5 in the temperate zone of Europe. The native American is more precocious than the American born of foreign parents, but the latter closely approximates the American of American parentage, even in the first generation.

Racial characteristics fade rapidly away; the age of puberty in Germany is 15.5 to 16; in Ireland, 15.3, and for the girls born in America of German or Irish parentage, 14.5, in St. Louis as it is in Montreal. The Canadian French are the only exception; between 14 and 15 in their native land, these alone are more precocious than the American of the same class when born in this country. 13.7 is the mean age; climate here has absolutely no influence; race very little; mentality, surroundings, education and nerve stimulation stand out prominently in this country as the factors which determine precocity.

